# Twenty-Seventh Annual Meeting of the American Medical Association

**Published:** 1876-06

**Authors:** 


### The Twenty-seventh Annual Meeting of the American Medical Association.

The twenty-seventh annual session of the American Medical Association was held during June 6, 7, 8 and 9, at Horticultural Hall, Philadelphia. At eleven o'clock on the morning of Tuesday, June 6, the meeting came to order, the President, Dr. J. Marion Sims, occupying the chair, the Vice-Presidents, Drs. Samuel Lily, of New Jersey, Wm. Pinkney, U. S. N., and S. D. Seelys, of Alabama, being upon the platform. Rev. Dr. E. R. Beadle offered a prayer, at the conclusion of which a graceful and eloquent welcome was extended to the visiting delegates by Dr. Wm. Pepper, Chairman of the Committee of Arrangements.

The President then read his address. After congratulating the delegates on the privilege of meeting here in this Centennial year and joining in the Centennial celebrations, the speaker said that this city is particularly interesting to this Association, for here it, twenty-nine years ago, was organized. Its first meeting was held in Baltimore, in 1848, when the beloved Chapman was elected President.

The speaker then traced the progress of the society, alluding to the last meeting in this city in 1872, when so much time was lost in the discussion of the woman question. But now, if any woman, entering the medical profession, rises to such a position as to be sent as a State or County delegate, the Association is bound to receive her; and so too with colored physicians.

The President then entered upon the discussion of the various forms of medical education in this country, and the code of ethics.

The three points in the code of ethics upon which he especially dwelt were, the portions relating to advertising, to the patenting of medical instruments, and to the secrecy of the consultation-room. He did not absolutely affirm as his desire that all restrictions upon advertising should be abolished, but certainly led his hearers up to the surmise that such abolition would, in his opinion, be best. He called attention to the arbitrary nature of the lines drawn, and to their variance in different countries and at different times in the same country. Thus, not many years since, a physician who put his office-hours upon his window-sill would have been sent to Coventry by his compeers; but now the practice has become common. Again, in the United States, every physician puts his name and occupation upon his window and door, but none dare advertise; whilst in France the doctor is prohibited from marking his door or window by a sign-board, but is allowed to advertise in other methods. Upon the hardship of the denial of the right of the physician to obtain patents upon instruments, Dr. Sims dwelt at considerable length. He cited instances where men had worked in the face of poverty and of all manner of discouragements for years, and their success when it had finally been achieved all went, not for the good of the inventors' families, not for the good of the sick and suffering patients, but for the building up and enriching of the—instrument-maker. He detailed the case of a friend of his who, broken in health, without means for supporting his family, had asked what he should do. The answer, which is at present being acted upon, was, "Leave the profession; become an instrument-maker; it is better than to stay in the profession and attempt to withstand public opinion, unjust and outrageous although it be." Patent and proprietary medicines, such as McMunn's Elixir, are used in large amounts by members of the regular profession in good standing, ay, by the very men who would thus deprive the medical inventor of the right possessed by every cursed son of Adam,—to eat bread in the sweat of his brow. Privacy in consultation was denounced by Dr. Sims as a thing which eqisted in name rather than in reality, and which ought not to exist at all. An honest man, he thought, would never have reason for hiding that which he had done and said.

On the whole the code is not up to the standard of professional honor, and at the same time hampers the profession in many ways. It is violated every day, not only by the rank and file, but also by those high in authority; and who dare call them to account? This is the first time that the validity, the constitutionality, of the code has been questioned; but he did not think it worth while that a committee should be appointed to investigate and alter it. Let the code stand as it is, but let it become a dead letter; honorable men do not need it to influence their actions; dishonorable men will not regard it or any other. Let it be the object of the American Medical Association to educate its members up to that higher code, that unwritten law, which is the universal standard of England.

The speaker then discussed syphilis, and urged with great emphasis and force the desirability—more than that, the necessity—for legislation which shall meet and combat this terrible scourge in a scientific and sufficient manner. Some startling statistics regarding prostitution on the Pacific Coast were given; and the speaker concluded his remarks on this subject by declaring that never must the evil be fought by licensing prostitution, and thereby upholding and cherishing vice, as obtains in Europe; a declaration which was received with loud applause.

The plan he had to suggest was one which did not recognize vice, and which yet would do away with the scourge syphilis. The sea is the great highway for syphilis, and sailors are the great carriers of the poison. A strict quarantine should be enforced; every steerage passenger and every sailor should be rigidly inspected, and, when the disease is found upon them, should be quarantined.

We want a system which will prevent the importation of disease from abroad, as well as one which will prevent its spread in our midst. The boards of health in cities, suggested the speaker, should be given the same powers to ferret out and to treat diseases of this nature as they now have in dealing with cholera or small-pox.

In conclusion, Dr. Sims said, in this our Centennial year, let us put far from us all sectional feelings, have nothing to do with any outside issues, and confine ourselves to the strict business of the Association.

The following day, after the appointment of a committee on examinations, and a report from the Judicial Council, Dr. R. C. Kedsie, of Lansing, Michigan, read a paper on Natural Purifiers, the first and most important of which, he contended, were air and water, oxygen being the essential element. He offered the following resolutions, which were adopted:

"*Resolved*, That it is the first duty of States and municipalities —first in importance and first in the order of time to make a sanitary survey of the water-supply, to preserve it against all unnecessary and avoidable contamination.

"*Resolved*, That no municipality should introduce a water-system without at the same time providing a corresponding and extensive sewer-system."

Dr. A. Garcelon, of Maine, then read a paper on Surgery, which was referred to the Committee on Publication.

Dr. Edward Seguin made the following report, with a resolution attached, which was adopted, in the name of a previous committee:

"Since several years, the American Medical Association has given its support to a measure of great interest for those who have at heart the advance of physic, namely:

"The establishment of uniform means of observation, and of medical records, for the physicians of all countries.

"This action of the American Medical Association has been expressed by the adoption of successive resolutions, and by the sending of delegates charged with the mission of advocating this reform:

"In 1873, to the British Medical Association, meeting in London; and to the French Association for the Advancement of the Sciences, meeting at Lyons.

"In 1874, to the British Medical Association, meeting at Norwich; and to the French Association for the Advancement of the Sciences, meeting at Lille.

"In 1875, to the International Medical Congress, meeting at Brussels.

"In 1876 next (September), the same Congress will meet in this very place; and now the American Medical Association is called to decide what position it will assume in this matter.

"Will it recede from its former position, and leave the task to second-hand promoters, or will it continue its initiative before the International Council?

"This is not only a question of pride for the Association, it is also one of justice due to the American physicians at large. If the constitution and by-laws of this Association prescribe an annual transfer of its meetings from one part to another of this vast country, it is to give us opportunities to study and express the wants of the whole profession. Of these wants, none has been found to be more deeply felt than the desire of partaking, as givers and receivers, in the discoveries of our art. But this want is not ours alone; it is universal; and if it succeed, the American Medical Association will deserve the thanks of all for having planned, and carried into execution, the most important instrument of internationalization of medical progress.

Therefore the Association should resolve to charge its delegates of former years to continue to advocate the uniformity of means of observation before the various medical societies, and particularly at the next International Medical Congress, and to report next year what success they may have met."

Drs. Seguin and Bowditch were appointed additional delegates to the International Congress.

A letter was read which had been received from the Medical Society of Victoria, asking for a list of the medical colleges of the United States recognized by the American Medical Association. This gave rise to a very active debate, attended with the wildest confusion, the President being completely unable to control the Association, and at times seemingly being forgetful of all parliamentary rules. Dr. Toner, of Washington, offered a resolution to lay the subject on the table,—which was carried unanimously.

On the next day, Thursday, June 8, after the reading of an excellent paper on Obstetrics, by Dr. S. C. Busey, of Washington, D. C., the roll was read and confirmed, various trifling exceptions being taken to certain names. On the reading of the name of Sarah Hackett Stevens, representing the Illinois State Society, Dr. Brodie, of Detroit, moved that that and all such names be referred to the Judicial Council. A motion that this resolution be laid upon the table was carried by a large vote, amidst considerable applause; and Sarah H. Stevens was therefore received as a delegate.

Dr. Henry A. Martin, of Boston, offered a resolution that the subject of bovine or animal vaccination is an important one as compared with the usual arm-to-arm practice, and that a committee be appointed to report upon the subject at the next meeting of the Association.

On Friday, June 9, the fourth and last day of the meeting, Dr. Toner, of Washington, offered the following resolution, which was unanimously adopted:

"*Resolved*, That members of the medical profession who in any way aid or abet the graduation of medical students in irregular or exclusive systems of medicine are deemed thereby to violate the spirit of the ethics of the American Medical Association."

Dr. H. C. Wood, of Philadelphia, offered the following, which was adopted:

"*Resolved,* That a committee of three be appointed by the Chair, to obtain from Congress an appropriation for the publication of the Subject Catalogue of the National Library, and that the State societies are requested to take such action as may be deemed fit to further said object."

The Committee on Nominations presented their report, which was adopted.

For President.—Dr. Henry J. Bowditch, of Massachusetts.

Vice-Presidents.—Dr. N. J. Pittman, of North Carolina; Dr. Franklin Staples, of Minnesota; Dr. Joseph R. Smith, of United States Army; Dr. Samuel C. Busey, of Washington, D. C.

Treasurer.—Dr. Casper Wistar, of Pennsylvania.

Librarian.—Dr. William Lee, of the District of Columbia.

Committee on Library.—Dr. Johnson Eliot, of the District of Columbia.

Assistant Secretary.—Dr. J. H. Hollister, of Illinois.

Committee of Arrangements.—Drs. N. S. Davis, J. W. Freer, H. A. Johnson, T. D. Fitch, H. Jones, Joseph P. Ross, and Leslie Curtis.

Committee of Publication.—Dr. W. B. Atkinson, Chairman; Drs. T. M. Drysdale, Albert Fricke, Samuel D. Gross, Casper Wistar, Richard J. Dunglison, all of Pennsylvania, and Dr. Milham, of the District of Columbia.

Medical Council.—Drs. N. S. Davis, of Illinois; E. L. Howard, of Maryland; W. O. Baldwin, of Alabama; H. W. Bean, of New York; A. N. Talby, of South Carolina; J. R. Logan, of Georgia; D. W. Stormont, of Kansas. This council is to take the place of the seven whose terms expire at this meeting. The rest of the present council is continued.

The time and place of the next meeting were fixed for the first Tuesday in June, 1877, at Chicago, Ill.

Dr. Bell, of Iowa, offered the following resolution, which was adopted:

"*Resolved,* That there be appointed a committee of three persons, members of this Association, in each of those States where there has been no action taken for the establishment of boards of health, to urge upon those States the necessity of the establishment of such boards."

The hour for adjournment having arrived, the newly-elected President, Dr. Bowditch, was conducted to the platform, when Dr. J. Marion Sims, the retiring President, read his farewell address. He said, "The moment draws near when we must say farewell. The events of this session will soon become history. In after-years we shall doubtless note changes in our organic laws that may be traced back to this meeting."

In closing, the doctor, in speaking of the Southern delegation, said, "With returning prosperity, our Southern brethern will be found working together shoulder to shoulder with us. Where once there was bitterness and hate, there is now peace and love. We have risen above all sectional feelings, which is as it should be; and now, in this Centennial year, Massachusetts and South Carolina can join hands in this peaceful hall of science." The doctor here turned to the newly-elected President and clasped his hand,—which action was vociferously applauded. The speaker then introduced the new President to the Convention, saying,—

"May the blessing of heaven rest upon his head, on our whole country, and upon every member of this Association."

Dr. Bowditch then, in a brief address, returned thanks to the Convention for the honor conferred upon him, hoping that the pleasant relations now existing would continue.

Dr. E. R. Squibb offered the following preamble and resolution:

*Whereas,* The usual time for a decennial revision of the United States Pharmacopœa is drawing near; and

*Whereas,* The plan of revision and publication in force in 1870 may not now be the best that could be devised; therefore,

"*Resolved,* That the American Medical Association take the whole subject of the National Pharmacopœa into consideration, for a review of its management; and, for the present time, with especial reference to the following questions:

"First, Whether the present plan of decennial revision and publication be practically sufficient for the needs of the Materia Medica and Pharmacy of the present time; and, if not sufficient, whether a plan could be devised which might offer probable advantages enough to justify an attempt to disturb the present one.

"Second, Whether this Association be the proper custodian in this country of the interests involved in the National Pharmacopœia; and, if it be the proper source of the National Codex, whom can it invite to co-operate with it in the work?

"Third, If it be a work for this Association, in what way can its details be wisely undertaken with any prospect of material improvement upon the present plan?

"*Resolved,* That in order to facilitate mature and general deliberation upon so important a subject, the final discussion of these resolutions be laid over for at least one year, and that the matter be recommended to the President of the Association for consideration in his annual address to the meeting of 1877."

The resolutions were adopted as read, and the Convention adjourned *sine die.—Philadelphia Med. Times.*

